# Association between Ambient Temperatures and Mental Disorder Hospitalizations in a Subtropical City: A Time-Series Study of Hong Kong Special Administrative Region

**DOI:** 10.3390/ijerph15040754

**Published:** 2018-04-14

**Authors:** Emily Y. Y. Chan, Holly C. Y. Lam, Suzanne H. W. So, William B. Goggins, Janice Y. Ho, Sida Liu, Phoebe P. W. Chung

**Affiliations:** 1 The Jockey Club School of Public Health and Primary Care, Faculty of Medicine, The Chinese University of Hong Kong, Prince of Wales Hospital, Shatin, Hong Kong, China; hollylam@cuhk.edu.hk (H.C.Y.L.); wgoggins@cuhk.edu.hk (W.B.G.); janice.ho@link.cuhk.edu.hk (J.Y.H.); kevin.liu@cuhk.edu.hk (S.L.); phoebechung@cuhk.edu.hk (P.P.W.C.); 2 Nuffield Department of Medicine, University of Oxford, Oxford OX3 7BN, UK; 3 Department of Psychology, The Chinese University of Hong Kong, Hong Kong, China; shwso@psy.cuhk.edu.hk

**Keywords:** mental disorders (MD), hospitalization, temperature, time-series, subtropical city

## Abstract

*Background*: Mental disorders have been found to be positively associated with temperature in cool to cold climatic regions but the association in warmer regions is unclear. This study presented the short-term association between temperatures and mental disorder hospitalizations in a subtropical city with a mean annual temperature over 21 °C. *Methods*: Using Poisson-generalized additive models and distributed-lagged nonlinear models, daily mental disorder hospitalizations between 2002 and 2011 in Hong Kong were regressed on daily mean temperature, relative humidity, and air pollutants, adjusted for seasonal trend, long-term trend, day-of-week, and holiday. Analyses were stratified by disease class, gender and age-group. *Results*: 44,600 admissions were included in the analysis. Temperature was positively associated with overall mental-disorder hospitalizations (cumulative relative risk at 28 °C vs. 19.4 °C (interquartile range, lag 0–2 days) = 1.09 (95% confidence interval 1.03, 1.15)), with the strongest effect among the elderly (≥75 years old). Transient mental disorders due to conditions classified elsewhere and episodic mood disorders also showed strong positive associations with temperature. *Conclusion*: This study found a positive temperature–mental-disorder admissions association in a warm subtropical region and the association was most prominent among older people. With the dual effect of global warming and an aging population, targeted strategies should be designed to lower the disease burden.

## 1. Introduction

The earth’s surface and atmosphere are warming unequivocally and successively. According to the Intergovernmental Panel on Climate Change, estimated average temperatures increased by 0.78 °C (95% confidence interval (CI): 0.72 to 0.85 °C) between the 1850–1900 and 2003–2012 periods [1]. Although the global human-health burden of climate change has not been well quantified, temperature-related morbidity and mortality, particularly for heat effects, have been reported [2]. Mental illness is a global health burden accounting for 32.4% of years lived with disability (YLDs) and 13.0% of disability-adjusted life years (DALYs) [3]. Mental disorders also have substantial impacts upon suicide rate [4,5]. The World Health Organization estimated the prevalence of lifetime mental disorder (including anxiety, mood, externalizing, and substance use) to be within the inter-quartile ranges of 18.1%–36.1% [6]. In Hong Kong, the estimated prevalence of common mental disorders among Chinese adults aged 16–75 years was 13.3% while the prevalence of severe non-specific psychological stress was 4.8% among the general population aged 18–64 years [7].

The number of studies looking at the association between mental disorders and ambient temperature have been increasing in recent years. Studies from Shanghai and Toronto [8,9], two Northern Hemispheric cities located at high latitudes above the Tropic of Cancer (23°26′12.9″), have reported higher relative risks of mental-disorder admissions at warmer temperatures, but no association at low temperatures. A majority of other studies also reported a higher risk of mental disorders during warmer temperatures but they focused on hot seasons only [10,11], were conducted during heat waves [12,13,14], looked at the effect of seasonality [15,16], used statistical methods not capable of revealing non-linear and lagged effects [17,18], or were not population-based [19]. Suicide rate, which is relevant to mental disorders, has also been found increasing with warmer temperatures [20,21,22] in different regions.

Although several studies have reported the effects of temperature on mental disorders, analyses of threshold temperatures, lagged effects, and relative risk were seldom included. Our team has studied the temperature–health association in Hong Kong, a highly dense subtropical city, and has already found heterogeneous associations of high temperature with the risk of various health problems, including cardiovascular diseases [23,24,25], respiratory diseases [26,27], infectious diseases [28], and overall hospitalization and mortality [29,30,31]. With increasing global average temperatures, understanding the potential impact of high temperatures on mental disorder hospitalizations will support the development of targeted population-based health policies that address the needs of mental health patients. This study aimed to evaluate the short-term association between ambient temperatures and mental-disorder hospitalizations in a subtropical city with a mean annual temperature over 21 °C.

## 2. Materials and Methods

### 2.1. Study Population and Data Collection

Hong Kong is located between Latitude 22°08′ N and 22°35′ N, Longitude 113°49′ E and 114°31′ E, with a population size of 7.07 million in mid-2011 and a subtropical climate (humid summer and mild winter). This study used public-hospital admissions data from the Hospital Authority of Hong Kong, which accounts for about 83% of the city’s overall hospitalizations [29]. Specifically for mental disorders, more than 99% of cases are admitted to public hospitals [32]. The daily number of hospital admissions between 2002 and 2011 with principal diagnosis at discharge as mental disorders (MD) (International Classification of Diseases ICD-9-290.xx-319.xx) was obtained. ICD-9 was used in this study, as the clinical record system of the Hospital Authority in Hong Kong had kept record using ICD-9 during the study period. In addition to information on diagnosis, each admission record contained details on patient age and gender and all data was kept anonymous. Daily meteorological records measured at the Hong Kong Observatory (HKO) were obtained for the study period, including mean temperature and mean relative humidity (RH), from the open-access data available on their website (http://www.hko.gov.hk/cis/climat_e.htm). The HKO data was selected because the website provides complete records for the required variables with no missing data during the study period and the monitoring station is located near the center of Hong Kong, enabling recorded temperatures to be representative of the general population. Aside from temperature, high levels of air pollutants have been reported to be associated with a higher risk of mental disorders [33,34,35,36,37,38,39]. Daily air pollutant levels were also included in models to assess the associations and account for their potential confounding effects. Daily average level of air pollutants from all general air quality monitoring stations except Tap Mun were downloaded from the official website of the Environmental Protection Department of Hong Kong and averaged, including particulate matter (PM_10_), sulphur dioxide (SO_2_), nitrogen dioxide (NO_2_), and ozone (O_3_). Tap Mun station was excluded because it is located in a remote rural area with very low population density, thus the exposure might not be representative of the general population.

### 2.2. Statistical Analysis

A combination of Generalized Additive Model [40] and Distributed Lagged Non-linear Model [41] for time series was used to examine the short-term association between ambient temperatures and mental-disorder hospitalizations. In the core model, the daily count of mental-disorder admissions was regressed over daily mean temperature with daily mean RH, long-term trend (day of study), seasonal trend, holiday effect, day-of-week effect, and same-day rainfall adjusted. Same-day rainfall was adjusted based on the theory that heavy rain will discourage people from seeking medical help. The same approach was applied in previous local temperature–hospitalization studies [23,24,25,26,27,28]. Air pollutants were added to the core model one-by-one to examine their independent association with mental-disorder hospitalizations and potential confounding effects on temperature–mental-disorder admissions associations. Only air pollutants showing association with mental disorders with a 95% confidence interval of relative risk at extreme exposure (vs. reference exposure) not including 1.00 and those demonstrating confounding effects were kept in the final model. The confounding effects were assessed by comparing the temperature–mental-disorder admissions associations with and without adjusting for air pollutants. As environmental variables, including temperature, RH, and air pollutants, may have a delayed effect (lagged effect) on health outcomes, the Distributed Lagged Non-linear Model, dlnm() package in R [41], was adopted for the potential lagged effects. Temperature, RH, and air pollutants were modeled as crossbasis() terms. The degrees of freedom (df) used for crossbasis terms [41] were chosen between 2 and 5 based on the robustness of association and the balance between computation cost and smoothing (by minimizing Generalized Cross Validation (GCV) score in the mgcv() package in R [40]). The maximum lag considered was based on the lagged effect observed in the RR-lag plots. The maximum df set for long-term trend (day-of-study), 10, and seasonal trend (day-of-year), 7, were based on the rule of thumb that a total of *n* × 7 df were allowed for smoothing a *n*-year trend. The basic formula was shown as below:

Log (E [daily no. of MD admissions]) = cb (temperature, df = 2–5; lag, df = 2–5) + cb (RH, df = 2–5; lag, df = 2–5) + cb (air pollutants, df = 2–5; lag, df = 2–5) + s (sqrt.Rain,k = 3) + s (DOS,k = 11) + s (DOY,k = 8) + factor (DOW) + factor (Holiday)

cb: crossbasis of independent variables built up with dlnm() package in R
s(): smoothing function of independent variables in mgcv() package in R
k: limitation of degrees of freedom in smoothing function (k-1 = max. df considered)
factor(): indicator of categorical independent variables
air pollutants: PM_10_, SO_2_, NO_2_ or O_3_
DOS: Day of study (1,2,3…,3227)
DOY: Day of year (1,2,3, …,365/366)
DOW: Day of week (1,2,3,…,7)

Subgroup analyses by gender and age-group (elderly ≥75 years, older adults 60–74 years, adults 15–59 years, and children <15 years) were performed to assess potential effect modification. Sub-disease classes—including persistent mental disorders due to conditions classified elsewhere (ICD-9 294.xx), dementias (ICD-9 290.xx), schizophrenic disorders (ICD-9 295.xx), episodic mood disorders (ICD-9 296.xx), other nonorganic psychoses (298.xx), anxiety, dissociative and somatoform disorders (ICD-9 300.xx), depressive disorder, not elsewhere classified (ICD-9 311.xx), transient mental disorders due to conditions classified elsewhere (ICD-9 293.xx), drug-related mental disorders (ICD-9 292.xx and 305.xx) and alcohol-related mental disorders (ICD-9 291.xx and ICD-9 303.xx)—were evaluated in subgroup analyses. Cumulative relative risks (RR) were estimated by comparing the admission risk between extreme exposure and reference exposure. The extreme and reference points for comparisons were chosen based on the association found between exposure and overall admissions. For linear associations, risk-of-hospitalizations was compared between the 75th percentile and 25th percentile of exposure (IQR range). For non-linear associations, risk-of-hospitalizations at the 97th percentile of exposure was compared to that at median exposure. The same extreme and reference values were adopted in all subgroups analyses. Df of 4 and 14 for the long-term time trend were used for sensitivity analysis. Partial autocorrelation function and residual plots were used for assessing appropriateness of models. The study was approved by the Survey and Behavior Research Ethics Committee of the Chinese University of Hong Kong.

## 3. Results

### 3.1. Descriptive Results

There were a total of 3652 days in the study period. Among the 7,150,288 all-cause admissions, there were 44,600 mental-disorders admissions (0.62%) and the daily mean number of mental-disorders admissions was 12.21. Among these, 52.45% were male, 36.19% were elderly aged ≥75 years, 16.39% were older adults aged 60–74 years, 45.55% were adults aged 15–59 years, and 1.87% were children aged below 15 years (Table 1). The breakdown of the sub-disease groups is in Table 2. The median daily mean temperature during the study period was 24.6 °C (IQR: 19.40, 27.80). The descriptive statistics for RH and the four air pollutants are shown in Table 3. 

### 3.2. Regression Results

**Figure 1:**
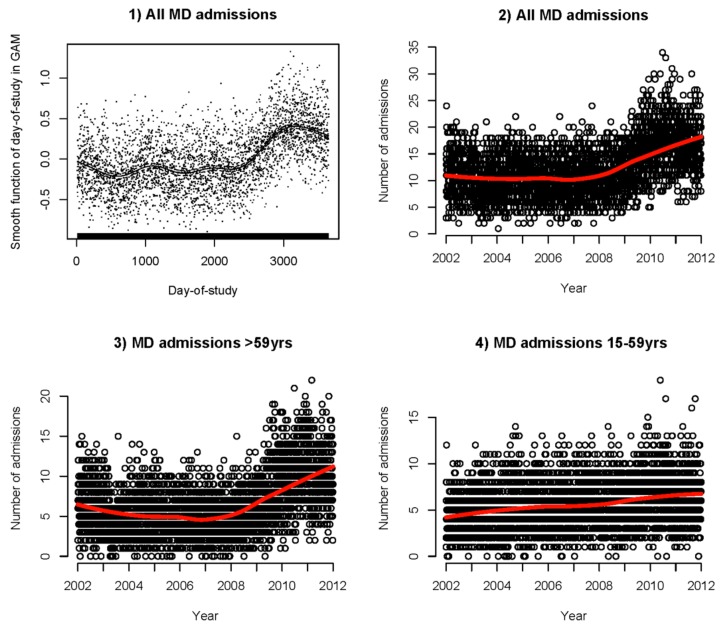
Long-term trend plot of mental-disorders admissions (**1**) after adjusting for temperature, relative humidity, NO_2_ level, seasonal trend, day-of-week, holiday effect, and same day rainfall, (**2**) long-term trend without adjustment for overall admissions, (**3**) long-term trend without adjustment for age >59 years and (**4**) long-term trend without adjustment for age below 60 years in Hong Kong, 2002–2011.

Among the four air pollutants studied, only NO_2_ showed a significant association with mental-disorder admissions. No confounding effects by PM_10_, SO_2_, and ozone were observed and therefore NO_2_ was the only air pollutant included in the final model. The df chosen for temperature and RH were 2 and that for NO_2_ was 3. The maximum lag considered was 10 days with the df of 4 for lagged effect. There was no significant seasonal trend for mental-disorder admissions before and after adjustment of environmental factors, but a sharp increase in the number of cases after March 2008 (around the 2250th day-of-study) was observed (Figure 1), regardless of the adjustment of environmental factors in the model. The increasing trend after 2008 was mainly driven by the age-group >59 years. Results of sensitivity analyses were consistent with those from the main analyses. The associations between mental-disorder admissions and temperature are reported below while the results for NO_2_ can be referred to in Appendix A.

### 3.3. Temperature

**Figure 2:**
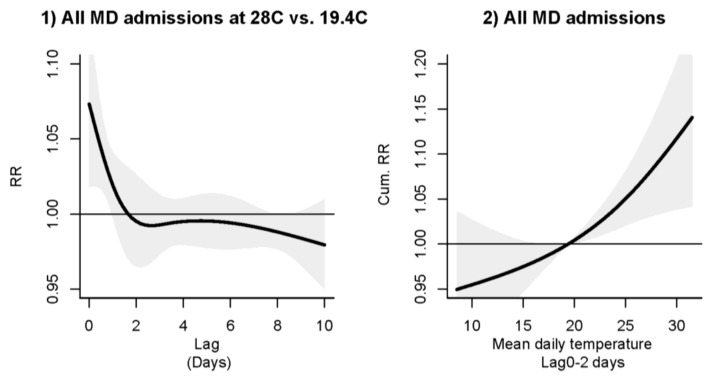
(**1**) left: RR-lag plot and (**2**) right: RR-temperature (°C) plot for overall mental disorders (MD) admissions, in Hong Kong, 2002–2011.

**Figure 3:**
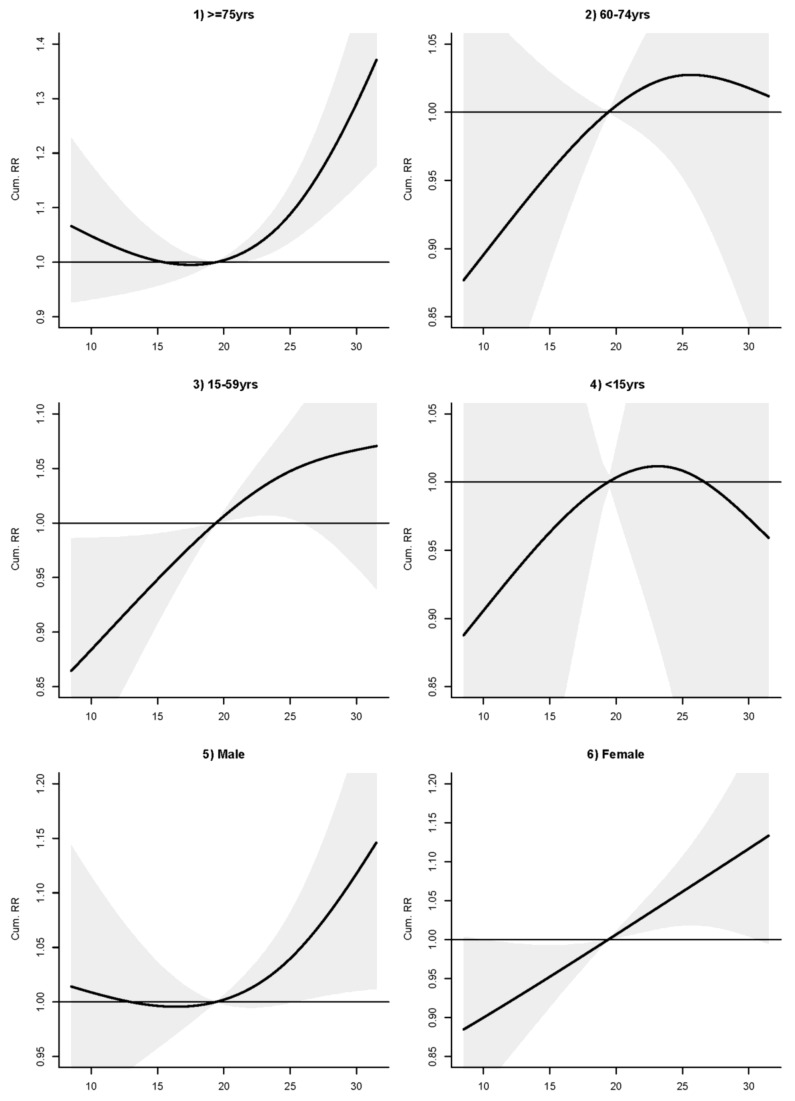
RR-temperature (°C) plots of mental disorder (MD) admissions in lagged 0–2 days for (**1**) elderly ≥75 years, (**2**) older adults 60–74 years, (**3**) adults 15–59 years, (**4**) children <15 years, (**5**) males and (**6**) females, Hong Kong, 2002–2011.

Temperature showed a positive linear association with mental disorders and the association lasted for about 2 days (Figure 2). The RR increased significantly when temperature rose over 19.4 °C (the lower quartile). The lagged 0–2 days RR at 28 °C (temperature at the 75th percentile vs. temperature at the 25th percentile at 19.4 °C) was 1.09 (95% confidence interval (1.03, 1.15)) (Table 4). Results in subgroup analyses showed that the linear association between temperature and overall mental-disorders admissions was mainly contributed by the adult group 15–59 years and the female group (Figure 2 and Figure 3), although the positive association for the 15–59 group became weaker when temperature was above 27 °C (with the lower confidence interval of RR dropping below 1.00). Above the threshold at about 20 °C, a strong association with warm temperatures were exhibited by the elderly group ≥75 years and males (Figure 3). The lagged 0–2 days RR at 28 °C (vs. 19.4 °C) for the elderly and adults were 1.20 (1.09, 1.31) and 1.06 (0.98, 1.15), respectively (Table 4). The lagged 0–2 days RR at 28 °C (vs. 19.4 °C) for males and females were 1.08 (1.01, 1.16) and 1.09 (1.01, 1.18), respectively. Temperature showed no obvious effects on older adults and children (Figure 3).

**Figure 4:**
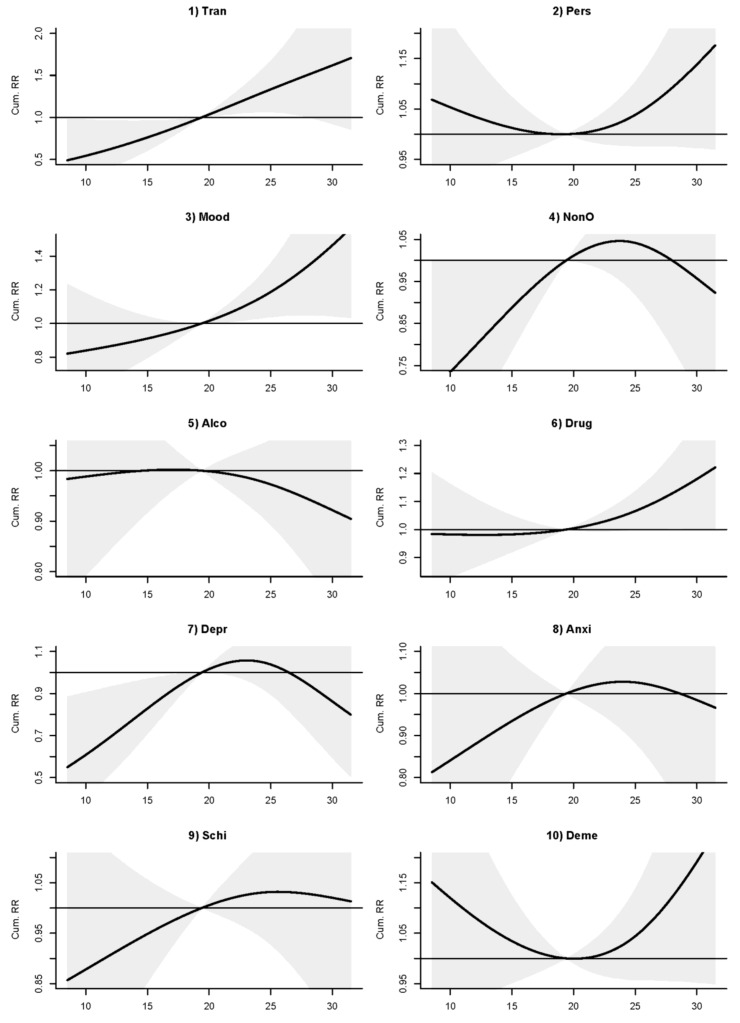
RR-temperature (°C) plots of mental-disorder (MD) admissions in lagged 0–2 days for (**1**) transient mental disorders (Tran), (**2**) persistent mental disorders (Pers), (**3**) episodic mood disorders (Mood), (**4**) other nonorganic psychoses (NonO), (**5**) alcohol-related mental disorders (Alco), (**6**) drug-related mental disorders (Drug), (**7**) depressive disorder (Depr), (**8**) anxiety, dissociative and somatoform disorders (Anxi), (**9**) schizophrenic disorders (Schi), and (**10**) dementias admissions (Deme) in Hong Kong, 2002–2011.

In sub-disease analyses, transient mental disorders and episodic mood disorders showed a positive association with temperature while drug-related mental disorders demonstrated a positive association when temperature rose over a threshold of about 20 °C (Figure 4). The association with transient mental disorders was strong and significant (RR 1.51 (1.00, 2.27)), followed in strength by episodic mood disorders (RR 1.34 (1.05, 1.71)), and then drug-related mental disorders (RR 1.13 (1.00, 1.27)) (Table 4). Depressive disorder and other nonorganic psychoses showed a significantly lower risk at lower temperature, while dementias and persistent mental disorders demonstrated a non-significant U-shaped association with temperature (Figure 4). No obvious association with temperature was observed for anxiety, dissociative and somatoform disorders, schizophrenic disorders, and alcohol-related mental disorders (Figure 4).

## 4. Discussion

Temperature was positively associated with mental-disorder admissions at lags 0–2 days. The association was mainly contributed to by females and adults 15–59 years. Transient mental disorders and episodic mood disorders demonstrated a robust positive association with temperature while drug-related mental disorders showed a positive association with temperatures above 20 °C. A sharp increase in the number of mental-disorder admissions was observed in 2008, particularly among older people above 60 years old. The reasons for the sharp growth in cases was unclear, but might be linked with an increasing awareness of Alzheimer’s, dementia, and cognitive problems [42] resulting from several non-governmental-organization-initiated health promotion programs targeting dementia in 2007 [7]. 

The results of this study were consistent with the two similar studies conducted at higher latitudes above the Tropic of Cancer. These studies, from Toronto [9] and Shanghai [8], found a higher relative risk of mental-disorder admissions at warmer temperatures with short lagged effect (less than 1 week) and observed no cold effect. As a subtropical city below Tropic of Cancer, the Hong Kong findings in this study demonstrated a linear relationship similar to Toronto but different from Shanghai, which had an obvious threshold. In contrast, a study from Germany found higher risks of all studied subtypes of mental disorders (ICD10 F00-F50) at lower temperatures (<10 °C) [43]. Several studies have evaluated the association between mental disorders and temperature during hot seasons [10,11] and heat waves [12,13,14], or compared the association between seasons [10], and most of these studies reported a higher risk at warmer temperatures. A study from Egypt reported a positive correlation with mania and a negative correlation with depression, but no association with schizophrenia [16]. Our findings and existing literature showed that admission-risk of mental disorders or related problems mostly increased as temperatures increased, regardless of geographic locations and latitude, and only a few studies have reported cold effects. 

Our study found stronger associations with temperature in the elderly group. This was consistent with most of the previous studies of a similar nature. Studies from Shanghai [8], Adelaide, Australia [10,12], Brisbane, Australia [44], and Vietnam [15] also reported a higher relative risk for the older group at warmer temperatures. Some studies, however, reported a higher risk among younger groups, including studies from Toronto [9] and Adelaide, Australia [13]. The higher relative risks found for the older age-group might be associated with a higher prevalence of cognitive problems among the older population. Previous studies have reported negative associations between temperature and cognitive function among Japanese [45] and Americans [46], while another study from Massachusetts, USA reported an U-shaped association with cognitive function and identified minimum risk at 10–15 °C [47]. Our study also found a U-shaped association between temperature and dementia admissions, although the association was nonsignificant. The study from Japan, however, did not find reduced cognitive function in increasing temperatures among men of other ethnicities, namely those of Southeast Asian descent, including Indonesians, Vietnamese, Thais, Filipinos, and Malaysians [45]. Although the sample size of that study was relatively small (32 individuals), and more work needs to be done to understand the association between temperature and cognitive function in different ethnicities, the authors suggested that the effect of high temperature affects people from higher latitudes more, which is a reasonable hypothesis when comparing the results among the Toronto study [9], the Shanghai study [8], and this Hong Kong study. Among the similar associations found in the three studies, the relative risk at 30 °C, when compared visually to 20 °C, was higher in Toronto, Latitude 43°42′ N, and Shanghai, Latitude 31°14′ N, than in Hong Kong, Latitude 22°35′ N. It seems that latitude could be correlated with the vulnerability to high temperature, although other regional factors like culture, policy, and behavior might also contribute to the differences. A point to note is that the risk of mental-disorder admissions at higher temperatures is still significant in Hong Kong, a subtropical city with annual mean temperature over 21 °C. This suggests that a similar risk may also be present in regions with latitude lower than 22°35′ N.

The results of this study showed that extreme high levels of nitrogen dioxide were associated with higher risk of overall mental-disorder admissions, and, in particular, transient mental disorders, anxiety, dissociative and somatoform disorders, persistent mental disorders, and schizophrenic disorders. However, due to the focus of this study on temperatures, the results for NO_2_ have been placed in Appendix A. Previous studies have reported significant associations of ambient air pollutants with mental disorders and the sub-diseases. Nitrogen dioxide is one of the air pollutants that has often been identified as an associating factor. Nitrogen dioxide, particulates, and ozone were found associated with higher odds of being diagnosed with autism among children in California [33,48]. PM2.5 was associated with anxiety symptoms among older female adults in the US [34]. Depression and related symptoms were found to be associated with exposure to high level of SO2, PM10, NO_2_, ozone, and CO in Korea [39,49], exposure to high level of NO_2_ in Barcelona [37] and Netherlands [35], but was negatively associated with NO_2_ in Norway [35]. Particulates were associated with schizophrenia hospitalizations and coarse particulates with overall mental-disorder hospitalizations in Beijing, China [38]. Studies from Sweden also reported an association between PM10 and overall psychiatric-emergency admissions in warm seasons [50] and found a positive association between NO_2_ and dispensed medications for psychiatric disorders in children and adolescents [51]. Our study agreed with most previous studies in finding that high NO_2_ levels were associated with mental disorders. In particular, NO_2_ has been reported to be affecting neurodegeneration [52] and this is consistent with our findings that the association with NO_2_ is most marked in the older age-groups. However, particulates, another pollutant that was commonly reported in other studies as an associating factor of mental disorders as well as reduced cognitive function [53], did not appear to be related to mental disorders in this study. More studies will be needed to investigate the biological mechanisms.

Ambient temperature can affect the risk of mental disorders in different ways. Temperature stress can affect physio-psychological functions directly by its effects on bio-chemicals. Heat stress is negatively associated with cognitive function and one of the plausible explanations is that it increases plasma serotonin which inhibits the production of dopamine, a neurotransmitter that is responsible for complex task performance [54]. Previous studies also reported a correlation between high temperature and altered platelet serotonin, which is associated with psychiatric disorders such as depression and schizophrenia [55,56]. High ambient temperature may also worsen the adverse health impacts on substance use, although patterns of substance use was not shown to change according to temperature. A study from New York found higher mortality from cocaine use during hot days (>31.1 °C) compared to other days [57], and hypothesized that extra stress added to the cardiovascular system by high temperature might worsen the existing cardiovascular conditions caused by cocaine use [57]. This was consistent with our results which found drug-related mental-disorder admissions were positively associated with temperature, although the association was not statistically significant. Other types of substance use that increase the cardiovascular load may cause similar effects and drive the number of hospital admissions.

In addition, previous studies have found increased mortality and morbidity risks at high temperatures among patients with mental and behavioral disorders [58,59]. People with reduced cognitive function may have less awareness of extreme environmental conditions and be less likely to apply self-protective measures, creating chances of higher personal exposures to high temperatures. Medications such as antipsychotics may also affect thermoregulation mechanisms like sweating, which may render patients with mental disorders more vulnerable to heat stress [58,59].

A previous review suggested that climate change affects mental illness mainly through the impact of natural disasters [60]. Although a single disaster event, such as heavy storms or flooding, will lead to a greater magnitude of direct and acute effects on mental health compared to a single heat wave or extreme high-temperature event, increasing temperature is an unavoidable exposure for the global population. Although the relative risk of mental-disorders admissions found in this study were not as strong as that of overall hospitalizations in our previous studies [29], the significant positive trend between temperature and mental disorders demonstrated that the sensitivity of mental disorders to temperature is not lower than other commonly studied heat-related disease groups. Furthermore, our study showed that age was an effect modifier of the temperature–mental disorder-admissions association in which mental-disorder admissions among the older population were more sensitive to high temperatures and high levels of NO_2_. The global aging population will certainly increase the size of this vulnerable group. With the dual effect of global warming and aging population, the burden of heat-related mental conditions will likely increase.

Policies promoting reduction of exposure to extreme high temperatures, such as reminders to use air conditioning and warning and outreach for services for susceptible groups, may be encouraged to lower the risk of mental-disorder hospitalizations in adults and elderly. On the other hand, NO_2_ is one of the traffic-related air pollutants, in which emission controls would be feasible. Air-quality policies reducing or limiting the release of NO_2_ should be promoted to protect the global population from excessive exposure to NO_2_. The lagged effects identified in this study, 0–2 days for temperature and 0–8 days for NO_2_, should also be considered by hospitals for capacity preparation.

This study has several limitations. First, this is an ecological study assuming the same exposures for the whole population. However, exposure levels may vary spatially, leading to an inaccurate estimation of relative risks for populations from different geographic locations. Furthermore, the temperature data for this study was from a single monitoring station. According to the regional weather report from HKO, regions that are farther from its station in the city center have a larger daily temperature range [61]. Therefore, this might underestimate the temperature exposure as well as the association of mental-disorder hospitalizations with temperature for populations living further away from the city center. Second, individual factors such as sociodemographic status, disease history, and drug history may be effect modifiers of the temperature–mental disorders-admissions association but were not available in this dataset. Third, this study assumed all admissions considered were directly caused by mental disorders and classified accurately. However, it is possible that patients were misclassified due to similar presentations of different disorders, or patients were admitted due to other symptoms but finally diagnosed as having mental disorder. Furthermore, as injury admission codes were not included in our study outcomes, any mental disorders leading to admissions of self-injury and suicidal classifications were not captured. Another limitation of this study is that we only had hospital-admissions data but did not have the data from the emergency department, and thus could not estimate the proportion of acute mental-disorder cases. Including only nonacute admissions in this study might have lowered the sensitivity of the analyses. This study also had some strengths. To the authors’ knowledge, this is the first study evaluating the short-term association between ambient temperature and mental-disorder hospitalizations in a region with a warm climate. Populations in warmer regions may have a higher burden of heat-related health problems, which may be additionally exacerbated by global warming. Although a similar study has been conducted in Shanghai, another subtropical city, it has a lower annual mean temperature than Hong Kong [62]. Additionally, the 10-year dataset used in this study provided adequate power to investigate the association between health outcomes and environmental exposure. The results of this study provide a good level of generalization since the dataset covers about 99% of mental-disorder hospitalizations in the population. The sophisticated statistical models used in the study also allowed for consideration of non-linear and lagged associations with confounders adjusted.

## 5. Conclusions

High temperature was significantly associated with an increased number of mental-disorder hospitalizations in a subtropical urban city with average annual temperatures above 21 °C. The association was stronger among people above 75 years old. With the collective effect of global warming and an aging population, the number of heat-related mental disorders may increase and may further intensify the disease burden across the public health, economic, and societal contexts. Health care professionals, patients, and caregivers should be advised to reduce exposure. Future studies for sub-diseases and understanding the biological mechanisms are warranted.

## Figures and Tables

**Table 1:** Descriptive statistics for mental-disorder admissions in public hospitals in Hong Kong, 2002–2011.

Group	Sub-Group	*N*	%
Total		44,600	100
Gender	Male	23,392	52.45
	Female	21,208	47.55
Age-group (years)	Elderly ≥ 75	16,142	36.19
	Older adults 60–74	7310	16.39
	Adults 15–59	20,315	45.55
	Children <15	833	1.87

**Table 2:** Distribution of mental-disorder hospitalizations by ICD-9 code (principal diagnosis at discharge), Hong Kong, 2002–2011.

ICD-9-Code	*N* (Total = 44,600)	%
294 Persistent mental disorders due to conditions classified elsewhere	10,085	22.61%
305 Nondependent abuse of drugs	5898	13.22%
290 Dementias	4896	10.98%
303 Alcohol dependence syndrome	3255	7.30%
295 Schizophrenic disorders	3218	7.22%
292 Drug-induced mental disorders	2586	5.80%
298 Other nonorganic psychoses	2270	5.09%
296 Episodic mood disorders	2076	4.65%
311 Depressive disorder, not elsewhere classified	1663	3.73%
309 Adjustment reaction	1570	3.52%
291 Alcohol-induced mental disorders	1556	3.49%
300 Anxiety, dissociative and somatoform disorders	1510	3.39%
293 Transient mental disorders due to conditions classified elsewhere	744	1.67%
307 Special symptoms or syndromes not elsewhere classified	667	1.50%
308 Acute reaction to stress	452	1.01%
304 Drug dependence	423	0.95%
297 Delusional disorders	380	0.85%
318 Other specified intellectual disabilities	314	0.70%
301 Personality disorders	260	0.58%
310 Specific nonpsychotic mental disorders due to brain damage	195	0.44%
319 Unspecified intellectual disabilities	129	0.29%
317 Mild intellectual disabilities	109	0.24%
313 Disturbance of emotions specific to childhood and adolescence	65	0.15%
306 Physiological malfunction arising from mental factors	57	0.13%
312 Disturbance of conduct not elsewhere classified	53	0.12%
302 Sexual and gender identity disorders	41	0.09%
315 Specific delays in development	40	0.09%
299 Pervasive developmental disorders	36	0.08%
316 Psychic factors associated with diseases classified elsewhere	33	0.07%
314 Hyperkinetic syndrome of childhood	19	0.04%

**Table 3:** Descriptive statistics for environmental variables in Hong Kong, 2002–2011.

Environmental Variables	Minimum	25th Percentile	Median	Mean	75th Percentile	Maximum
Daily mean temperature (°C)	8.20	19.40	24.60	23.45	27.80	31.80
Daily mean relative humidity (%)	31.00	73.00	79.00	78.02	85.00	98.00
Daily mean level of PM_10_ (µg/m^3^)	8.71	42.66	57.54	56.23	63.10	389.05
Daily mean level of NO_2_ (µg/m^3^)	18.95	42.79	54.78	56.75	68.51	146.61
Daily mean level of SO_2_ (µg/m^3^)	3.50	11.40	16.35	18.96	23.06	110.44
Daily mean level of O_3_	6.22	19.70	32.08	35.47	47.61	118.45

**Table 4:** Regression results for temperature with overall mental-disorders admissions and subgroup admissions in Hong Kong, 2002–2011.

Sub-Group	RR	95% Confidence Interval	
		Lower	Upper
Daily Mean Temperature Lagged 0–2 Days 28 vs. 19.4 °C (75th Percentile vs. 25th Percentile)			
Overall admissions	1.09	1.03	1.15
Males	1.08	1.01	1.16
Females	1.09	1.01	1.18
Elderly (≥75)	1.20	1.09	1.31
Older adults (60–74)	1.02	0.89	1.17
Adults (15–59)	1.06	0.98	1.15
Children (<15)	0.99	0.67	1.47
Persistent mental disorders	1.09	0.98	1.22
Dementias	1.12	0.96	1.32
Schizophrenic disorders	1.03	0.85	1.25
Episodic mood disorders	1.34	1.05	1.71
Other nonorganic psychoses	1.00	0.79	1.26
Anxiety, dissociative and somatoform disorders	1.01	0.76	1.32
Depressive disorders	0.95	0.72	1.24
Transient mental disorders	1.51	1.00	2.27
Drug-related mental disorders	1.13	1.00	1.27
Alcohol-related mental disorders	0.94	0.81	1.11

**Figure A1:**
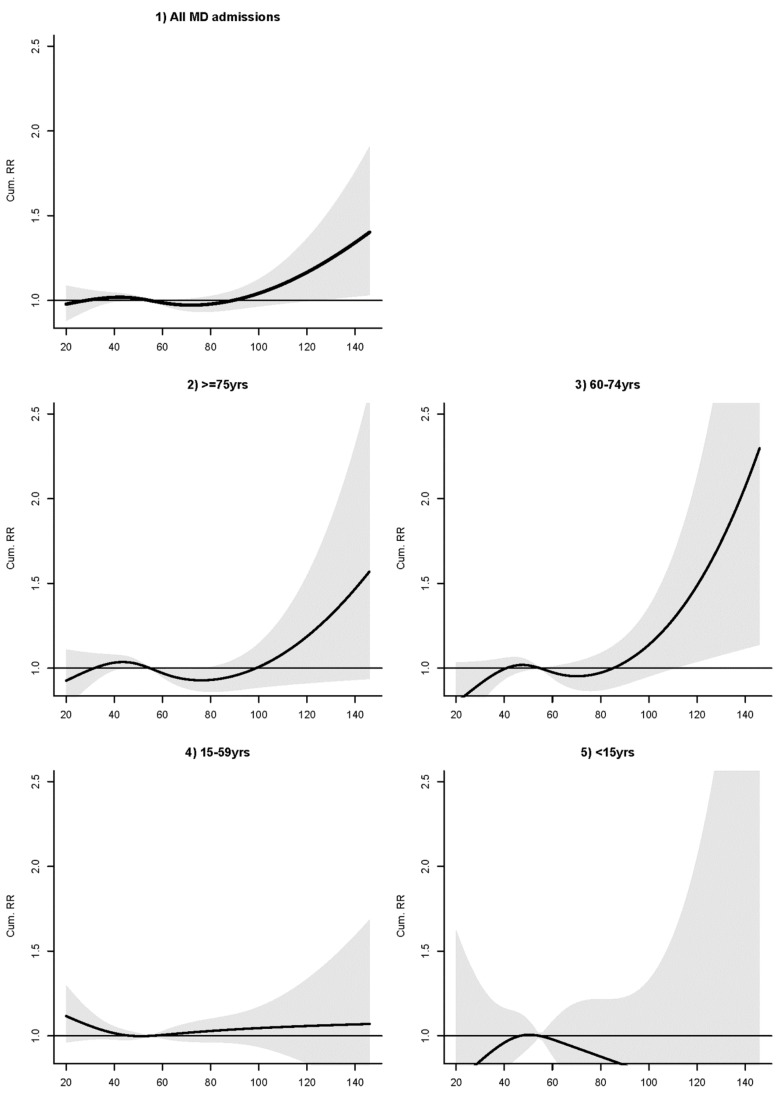
RR-NO2 (µg/m^3^) plots in lagged 0–8 days for (**1**) overall mental disorders (MD) admission, (**2**) elderly ≥75 years, (**3**) older adults 60–47 years, (**4**) adults 15–59 years and (**5**) children <15 years, in Hong Kong, 2002–2011.

**Figure A2:**
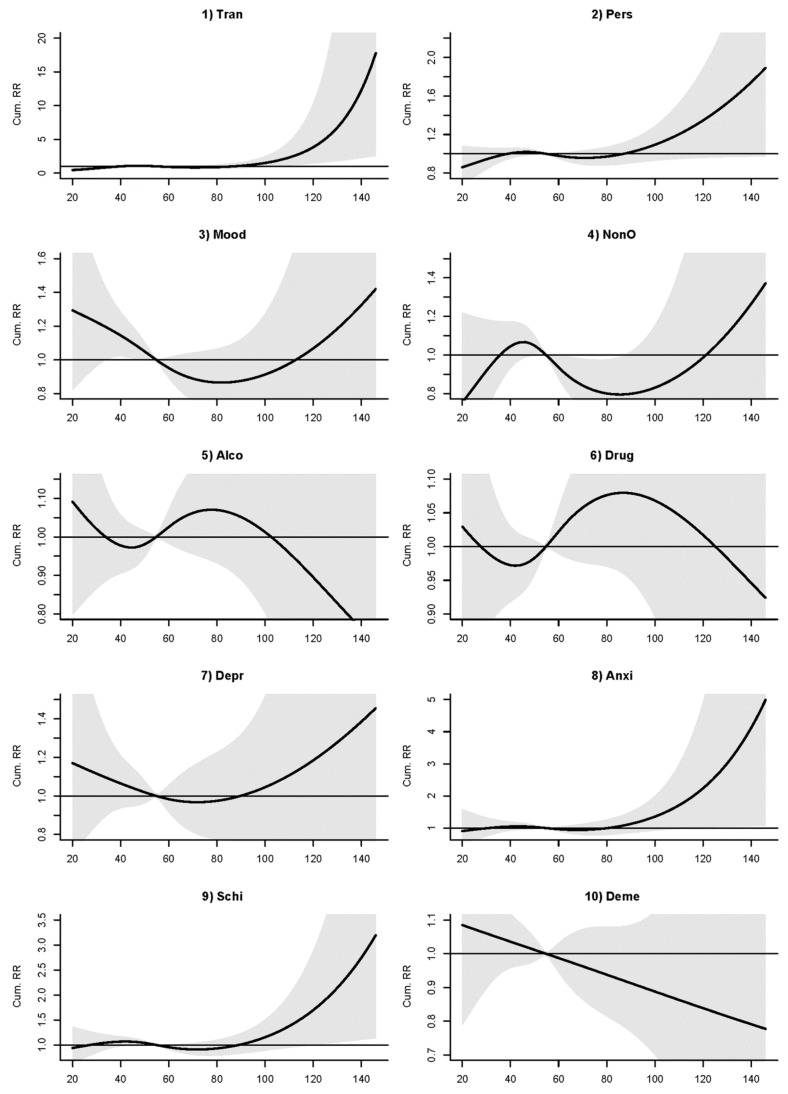
RR-NO2 (µg/m^3^) plots of mental disorder admissions in lagged 0–8 days for (**1**) transient mental disorders (Tran), (**2**) persistent mental disorders (Pers) , (**3**) episodic mood disorders (Mood), (**4**) other nonorganic psychoses (NonO), (**5**) alcohol-related mental disorders (Alco), (**6**) drug-related mental disorders (Drug), (**7**) depressive disorder (Depr), (**8**) anxiety, dissociative and somatoform disorders (Anxi), (**9**) schizophrenic disorders (Schi) and (**10**) dementias admissions (Deme) in Hong Kong, 2002–2011.

## Appendix A. Regression Result for NO_2_—the Short-Term Associaton between NO_2_ and Mental Disorder Admissions

### Nitrogen Dioxide NO_2_

Relative risks of admissions increased when NO_2_ level rose over 90 µg/m^3^, which is about the 95th percentile (Figure A1). The increased RR became significant when NO_2_ level was higher than 120 µg/m^3^ (~99.7th percentile). The increased risk at extreme high level of NO_2_ lasted for about 8 days. The lagged 0–8 days RR at the 99.7th percentile of NO_2_ (120 vs. median = 56 µg/m^3^) was 1.17 (1.00, 1.36) (Table A1). Subgroup analyses showed that the elderly ≥75 years and older adults 60–74 years were sensitive to high level of NO_2_, with significant association seen in the older adult groups (Figure A1). The lagged 0–8 days RR at high level of NO_2_ (120 vs. 56 µg/m^3^) for the elderly and older adults were 1.19 (0.91, 1.55) and 1.49 (1.04, 2.13), respectively. Females showed a slightly higher RR than males but the difference was not obvious.

Sub-disease analyses showed that an extreme high level of NO_2_ was associated with a higher admission risk of transient mental disorders, anxiety, dissociative and somatoform disorders, persistent mental disorders, and schizophrenic disorders (Figure A2 and Table A1). The associations with transient mental disorders (RR 3.83 (1.42, 10.31)) and anxiety, dissociative and somatoform disorders (RR 2.23 (1.02, 4.91)) were very strong. No obvious associations with NO_2_ were observed in other sub-disease categories (Figure A2).

**Table A1:** Regression results for NO2 with overall mental disorders admissions and subgroup admissions in Hong Kong, 2002–2011.

Sub-Group	RR	95% Confidence Interval	
		Lower	Upper
Daily Mean NO_2_ Level Lagged 0–8 Days 120 vs. 56 µg/m^3^ (99.7th Percentile vs. 50th Percentile)			
Overall admissions	1.17	1.00	1.36
Males	1.10	0.89	1.37
Females	1.23	0.98	1.53
Elderly (≥75)	1.19	0.91	1.55
Older adults (60–74)	1.49	1.04	2.13
Adults (15–59)	1.06	0.84	1.33
Children (<15)	0.68	0.23	2.05
Persistent mental disorders	1.35	0.96	1.89
Dementias	0.84	0.51	1.36
Schizophrenic disorders	1.70	1.00	2.88
Episodic mood disorders	1.07	0.54	2.09
Other nonorganic psychoses	0.99	0.50	1.94
Anxiety, dissociative and somatoform disorders	2.23	1.02	4.91
Depressive disorders	1.18	0.55	2.53
Transient mental disorders	3.83	1.42	10.31
Drug-related mental disorders	1.02	0.71	1.47
Alcohol-related mental disorders	0.90	0.55	1.45
